# Extensive Within-Host Diversity in Fecally Carried Extended-Spectrum-Beta-Lactamase-Producing Escherichia coli Isolates: Implications for Transmission Analyses

**DOI:** 10.1128/JCM.00378-15

**Published:** 2015-06-18

**Authors:** N. Stoesser, A. E. Sheppard, C. E. Moore, T. Golubchik, C. M. Parry, P. Nget, M. Saroeun, N. P. J. Day, A. Giess, J. R. Johnson, T. E. A. Peto, D. W. Crook, A. S. Walker

**Affiliations:** a Nuffield Department of Clinical Medicine, University of Oxford, Oxford, United Kingdom; b NIHR Biomedical Research Center, University of Oxford/Oxford University Hospitals NHS Trust, Oxford, United Kingdom; c Department of Clinical Research, London School of Hygiene and Tropical Medicine, London, United Kingdom; d School of Tropical Medicine and Global Health, Nagasaki University, Nagasaki, Japan; e Mahidol-Oxford Tropical Medicine Research Unit, Bangkok, Thailand; f Angkor Hospital for Children, Siem Reap, Cambodia; g Minneapolis Veterans Affairs Health Care System, Minneapolis, Minnesota, USA; h Department of Medicine, University of Minnesota, Minneapolis, Minnesota, USA

## Abstract

Studies of the transmission epidemiology of antimicrobial-resistant Escherichia coli, such as strains harboring extended-spectrum beta-lactamase (ESBL) genes, frequently use selective culture of rectal surveillance swabs to identify isolates for molecular epidemiological investigation. Typically, only single colonies are evaluated, which risks underestimating species diversity and transmission events. We sequenced the genomes of 16 E. coli colonies from each of eight fecal samples (*n =* 127 genomes; one failure), taken from different individuals in Cambodia, a region of high ESBL-producing E. coli prevalence. Sequence data were used to characterize both the core chromosomal diversity of E. coli isolates and their resistance/virulence gene content as a proxy measure of accessory genome diversity. The 127 E. coli genomes represented 31 distinct sequence types (STs). Seven (88%) of eight subjects carried ESBL-positive isolates, all containing *bla*_CTX-M_ variants. Diversity was substantial, with a median of four STs/individual (range, 1 to 10) and wide genetic divergence at the nucleotide level within some STs. In 2/8 (25%) individuals, the same *bla*_CTX-M_ variant occurred in different clones, and/or different *bla*_CTX-M_ variants occurred in the same clone. Patterns of other resistance genes and common virulence factors, representing differences in the accessory genome, were also diverse within and between clones. The substantial diversity among intestinally carried ESBL-positive E. coli bacteria suggests that fecal surveillance, particularly if based on single-colony subcultures, will likely underestimate transmission events, especially in high-prevalence settings.

## INTRODUCTION

*E*scherichia coli is considered an opportunistic pathogen of humans: although capable of causing a wide spectrum of clinical disease, it is carried asymptomatically in the gastrointestinal tract of >90% of individuals (1). The emergence and spread of antimicrobial resistance in E. coli is facilitated by its existence within the gut reservoir (2–4). The recent threat of broad-spectrum antibiotic resistance, such as that mediated by extended-spectrum beta-lactamases (ESBLs) (5), has prompted the introduction of fecal surveillance in an attempt to track drug-resistant organisms epidemiologically and deploy infection control measures to limit their spread (6, 7). Although ESBL-positive E. coli carriage prevalence varies from <10% to >70%, depending on the setting, it appears to be on the increase globally in all contexts, including in the community (8).

Current surveillance of the transmission of drug-resistant Enterobacteriaceae tends to focus on tracking the spread of individual clones (9), but whether this represents typical ESBL-positive E. coli carriage and transmission remains unclear. Our understanding of transmission is complicated by the fact that surveillance studies have used different approaches, varying in their (i) sampling time frames and strategies (7, 10, 11), (ii) methods of selective culture of drug-resistant colonies from rectal swabs (12), and (iii) phenotypic/molecular methods used to subsequently characterize strains (12, 13), with these methods themselves varying in their screening sensitivity and specificity. At odds with the single-colony approach is the fact that previous studies have demonstrated that the molecular diversity of E. coli bacteria carried in the gastrointestinal tract is likely to be substantial, both between and within individual human hosts (14, 15). Molecular typing of single clones may therefore be inappropriate for characterizing transmission, particularly in areas of high resistance prevalence where individuals may carry multiple distinct clones with the same resistance phenotype. Additionally, previous studies of intrahost strain diversity have investigated only short molecular targets, using techniques such as phylogrouping, random amplified polymorphic DNA (RAPD) analysis, or multilocus sequence typing (MLST) (6, 7, 14, 15), and may therefore have underestimated the extent of genetic diversity present.

Whole-genome sequencing (WGS) is a high-resolution typing technology that has been used successfully to describe within-host population structures of several bacterial pathogens, such as Staphylococcus aureus (16, 17). In this study, we used WGS as a single assay to sample genetic diversity in core and accessory genomes of E. coli in fecal samples taken from asymptomatic carriers in a community with high ESBL carriage prevalence (8).

(These data were presented in part at the 24th European Congress of Clinical Microbiology and Infectious Diseases, Barcelona, Spain, 10 to 13 May 2014 [18].)

## MATERIALS AND METHODS

### Sample description.

Fecal samples from eight randomly selected individuals, 13 to 14 years of age (considered to have adult fecal microflora), were investigated. These individuals were enrolled in a much larger study to estimate regional pediatric helminth prevalence (April to June 2012) undertaken at the Cambodia-Oxford Medical Research Unit (COMRU), Angkor Hospital for Children, in Siem Reap, Cambodia. To minimize microbial loss, the samples were stored immediately after collection as fecal slurries in saline with 10% glycerol at −80°C (19). The local hospital Institutional Review Board ([IRB] date of approval, 12 March 2012) and the Oxford Tropical Research Ethics Committee ([OXTREC] reference number 12-12) approved the wider research use of these samples.

### Laboratory methods.

For each fecal slurry, 5 μl of neat sample, and of 1:5 and 1:100 dilutions was streaked evenly across individual CHROMagar Orientation agar plates (Becton Dickinson UK, Oxford, United Kingdom) and then incubated in air at 37°C overnight after placement of a cefpodoxime disc (10 μg; Oxoid, Basingstoke, United Kingdom) on each plate. E. coli isolates are typically represented as pink colonies on CHROMagar Orientation agar.

For each sample, a dilution plate was selected on which growth was sparse enough to enable 16 single-colony picks of discrete pink colonies (presumptive E. coli), which were subcultured onto separate Columbia blood agar plates. These included up to eight colonies from within the inhibition zone around the antibiotic disc (if observed), with the balance taken from outside the inhibition zone. Plates were incubated overnight in air at 37°C.

### DNA extraction and sequencing.

DNA was extracted from the subcultures using a QuickGene kit (Fujifilm, Minato, Tokyo, Japan) according to the manufacturer's instructions, with an additional mechanical lysis step (FastPrep; MP Biomedicals, Santa Ana, USA) following the addition of chemical lysis buffer. Sequencing was carried out on an Illumina HiSeq 2500 platform, generating 100- or 151-base-paired end reads (see Table S1 in the supplemental material).

### Sequence read processing and data analysis.

Reads were mapped to the CFT073 E. coli reference genome (NCBI RefSeq NC_004431), and variants were called using a validated in-house bioinformatics pipeline (20). Reads were also assembled *de novo* using Velvet and the VelvetOptimiser wrapper (21, 22).

Multilocus sequence type (ST) and resistance/virulence gene content were determined *in silico* using BLASTn-based comparisons of reference alleles and *de novo*-assembled contigs for each isolate. For MLST we used the scheme and reference database of Wirth et al. (23); for resistance genes, an in-house database of curated resistance genes and variants (24) was used, and for virulence genes, a further in-house database compiled for this study from the Virulence Factors Database (VFDB) (25) and review papers (26, 27) was used. Virulence factors were subdivided into functional categories as follows: adhesins, toxins, nutritional, immune evasion-related, and miscellaneous (26). Multiple BLAST hits to overlapping positions on the same contigs were ignored, and only the best hit was retained; gene presence was arbitrarily defined as >80% sequence similarity over >80% of the length of the reference locus (i.e., coverage of the reference sequence of >80%).

Bootstrapped maximum likelihood phylogenies were derived from core chromosomal single nucleotide variants (SNVs; core SNVs defined as no null call in any isolate at that site from reference-based mapping) using PhyML (28). To scale trees appropriately, core SNVs for each isolate were reinserted into the reference CFT073 sequence, thereby generating an alignment of modified reference sequences of all the isolates for comparison.

Diversity was assessed first with respect to the core genome, using reference-mapped data, and second with respect to the small part of the accessory genome represented by the resistance/virulence genes described above. To avoid skewed results from repeated sampling of essentially the same organism, comparisons were made across all isolates and across all clones plus singletons. Here, a clone was defined as a set of at least two isolates from a given individual, where each isolate differed by no more than two core SNVs from at least one other isolate in the clone. No isolates had 3 to 10 SNV differences. Singletons were defined as any isolates that were not part of a clone. Our definition of a clone is independent of ST. The SNV cutoff defining a clone is arbitrary but conservative, given the extent of diversity observed in E. coli (29), and is consistent with SNV differences observed within hosts over 6 to 12 months for Clostridium difficile, another gastrointestinal organism with a similar mutation rate but far less intrahost diversity (30, 31). The accessory genome, represented here only by the particular resistance and virulence genes surveyed, was defined as homogeneous for a clone if every sequenced isolate within the clone had the same profile or as heterogeneous if these traits varied.

To assess whether genes present in the accessory genome were more likely to be similar across genetically distinct strains (represented here by clones and/or singletons [clones/singletons]) within an individual than strains between individuals, we generated a distance matrix from pairwise comparisons of the accessory gene content for each clone or singleton (as defined by the complete profile of resistance/virulence genes present in any isolate of that clone or singleton). The statistical significance of the difference in accessory gene diversity among strains within individuals versus strains between individuals was then calculated using a Wilcoxon rank sum test. This analysis tests the hypothesis that horizontal gene transfer of specific resistance/virulence genes is likely to be occurring with greater frequency within the gastrointestinal tract of individuals. Statistical analyses were carried out in Stata/SE, version 11.2 (StataCorp., Texas, USA).

### Microarray data accession number.

Data were deposited in the NCBI under BioProject number PRJNA274331. Details of sequenced strains and data accession numbers are provided in Table S1 in the supplemental material.

## RESULTS

Of eight samples, five came from female individuals; the median age was 13.4 years (interquartile range [IQR], 13.2 to 13.7 years). Six samples were obtained from children subsequently admitted to the inpatient department, and two were from patients reviewed in the outpatient department of the hospital (IHD45 and IHD717). Three children had underlying blood dyscrasias (thalassemia, subject IHD244; leukemia, subjects IHD232 and IHD813), and one had valvular heart disease (subject IHD1178). Details on previous antibiotic usage were not available; all individuals except subject IHD232 had exposure to either companion animals or livestock/poultry in the household.

The fecal cultures yielded presumptive cefpodoxime-resistant E. coli colonies within the peridisk inhibition zone for five of eight subjects (Table 1) (subjects IHD86, IHD244, IHD357, IHD813, and IHD1178). For these five subjects, simple random sampling of eight presumptive E. coli colonies from within and eight from outside the inhibition zone was undertaken (*n =* 16 per sample). For the remaining three subjects (IHD45, IHD232, and IHD717), the primary culture plates had no within-zone E. coli colonies, so 16 colonies were picked from outside the zone. The resulting total 128 colonies underwent genome sequencing, which failed for one isolate, leaving 127 genome sequences for analysis.

**TABLE 1:** Summary details on screening phenotype, sequence type, and CTX-M variants in study individuals

Subject (figure)	Phenotypic cefpodoxime resistance^*a*^	*bla*_CTX-M_ variant (proportion of isolates positive)	*bla*_CTX-M_-positive ST(s) (proportion of positive isolates per ST)	*bla*_CTX-M_-negative ST(s) (proportion of negative isolates per ST)	Diversity observed (no. of clones)^*b*^
IHD244 (Fig. 1A)	Yes	*bla*_CTX-M-14_ (8/16)	ST-226 (8/8)	ST-205 (1/1), ST-871 (7/7)	Only homogeneous clones (2)
IHD86 (Fig. 1B)	Yes	*bla*_CTX-M-14_ (2/15)	ST-10 (1/1), ST-2535 (1/2)	None	Same *bla*_CTX-M_ in different STs
		*bla*_CTX-M-55_ (13/15)	ST-2535 (1/2), ST-196 (5/5), ST-354 (6/6), ST-359 (1/1)		Same ST with different *bla*_CTX-M_ variants; homogeneous (1) and heterogeneous (2) clones
IHD232 (Fig. 1C)	No	*bla*_CTX-M-14_ (1/16)	ST-405 (1/1)	Novel (1/1), ST-2540 (1/1), ST-423 (3/3), ST-1286 (10/10)	Only heterogeneous clones (2)
IHD357 (Fig. 1D)	Yes	*bla*_CTX-M-15_ (8/16)	ST405 (8/8)	ST-46 (1/1), ST-4012 (7/7)	Homogeneous (1) and heterogeneous (1) clones
IHD45 (Fig. 1E)	No	*bla*_CTX-M-14_ (1/16)	Novel (1/1)^*c*^	ST-398 (1/1), ST-542 (1/1), ST-1684 (1/1), novel (2/2)^*c*^, ST-168 (2/2), ST-394 (2/2), ST-449 (2/2), ST-155 (4/4)	Homogeneous (3) and heterogeneous (1) clones; substantial nucleotide-level variation within ST-155
IHD813 (Fig. S1A)	Yes	*bla*_CTX-M-55_ (16/16)	ST-10 (16/16)	None	Single homogeneous clone
IHD1178 (Fig. S1B)	Yes	*bla*_CTX-M-15_ (16/16)	ST-405 (2/2), ST-648 (13/13), novel (1/1)	None	Same *bla*_CTX-M_ in different STs; only homogeneous clones (2)
IHD717 (Fig. S1C)	No	None	None	ST-3489 (1/1), ST-3759 (1/1), ST-155 (1/1), ST-939 (3/3), novel (10/10)	Homogeneous (1) and heterogeneous (1) clones; substantial nucleotide-level variation within ST-939

a Detected by disk screening.

b Homogeneous and heterogeneous refer to the accessory component of the genome, as represented by the profile of resistance/virulence gene presence/absence.

c Three different novel ST variants identified in this individual.

The median number of high-quality reads available for mapping per isolate was 3,159,616 (range, 1,510,888 to 4,777,836). This provided >70% coverage of the reference genome (median coverage, 73%; range, 71 to 79%), consistent with all isolates being E. coli. The median *de novo* assembly size was 4,818,355 bp (range, 4,513,031 to 5,306,174 bp), with a median 187 contigs (range, 76 to 422).

### *In silico* multilocus sequence typing.

*In silico* MLST identified BLAST matches for all E. coli housekeeping loci in all 127 isolates, including novel *fumC*, *purA*, and *recA* variants (one each). In total, the 127 sequenced genomes represented 31 STs (of which 6 were novel). Across all study participants, 18 clones were identified (collectively comprising 108 isolates), whereas 19 isolates were singletons, representing 18 different STs.

The STs were distributed unevenly across the eight fecal samples, which contained 1 to 10 (median, 4) STs each. ST-405 was carried by three individuals, and ST-10 and ST-155 were carried by two individuals each. The other STs were all identified in a single individual each (Table 1). Despite analysis of 16 colonies per subject, the 37 clones plus singletons were also unevenly distributed across the eight samples, which had 1 to 11 (median, 4) clones plus singletons each.

### Determination of genotypes accounting for cefpodoxime resistance.

A *bla*_CTX-M_ variant was detected in all 40 presumptive cefpodoxime-resistant isolates picked from within the peridisk cefpodoxime inhibition zone for the five samples with such colonies; this presumably explained these samples' cefpodoxime-resistant phenotypes. Additionally, for two of the three samples that yielded no colonies within the cefpodoxime inhibition zone, a *bla*_CTX-M_ variant was detected in 1/16 colonies (per sample) picked from outside the inhibition zone. Thus, at least 7/8 (88%) individuals carried *bla*_CTX-M_ genes. In two individuals (subjects IHD86 and IHD1178), the same *bla*_CTX-M_ variant was observed in genetically distinct isolates. Specifically, in subject IHD1178, *bla*_CTX-M-15_ was found in three clones plus singletons, whereas in subject IHD86, *bla*_CTX-M-55_ was found in four different clones plus singletons, and *bla*_CTX-M-14_ was found in 2 clones plus singletons. Furthermore, in IHD86, both *bla*_CTX-M-55_ and *bla*_CTX-M-14_ were observed within the same clone (ST-2535) (Table 1).

### Analysis of within-host E. coli core and accessory genome diversity.

**FIG 1:**
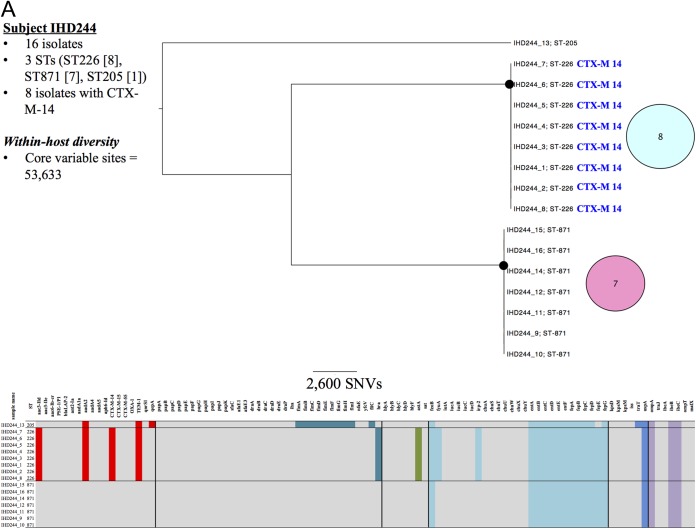

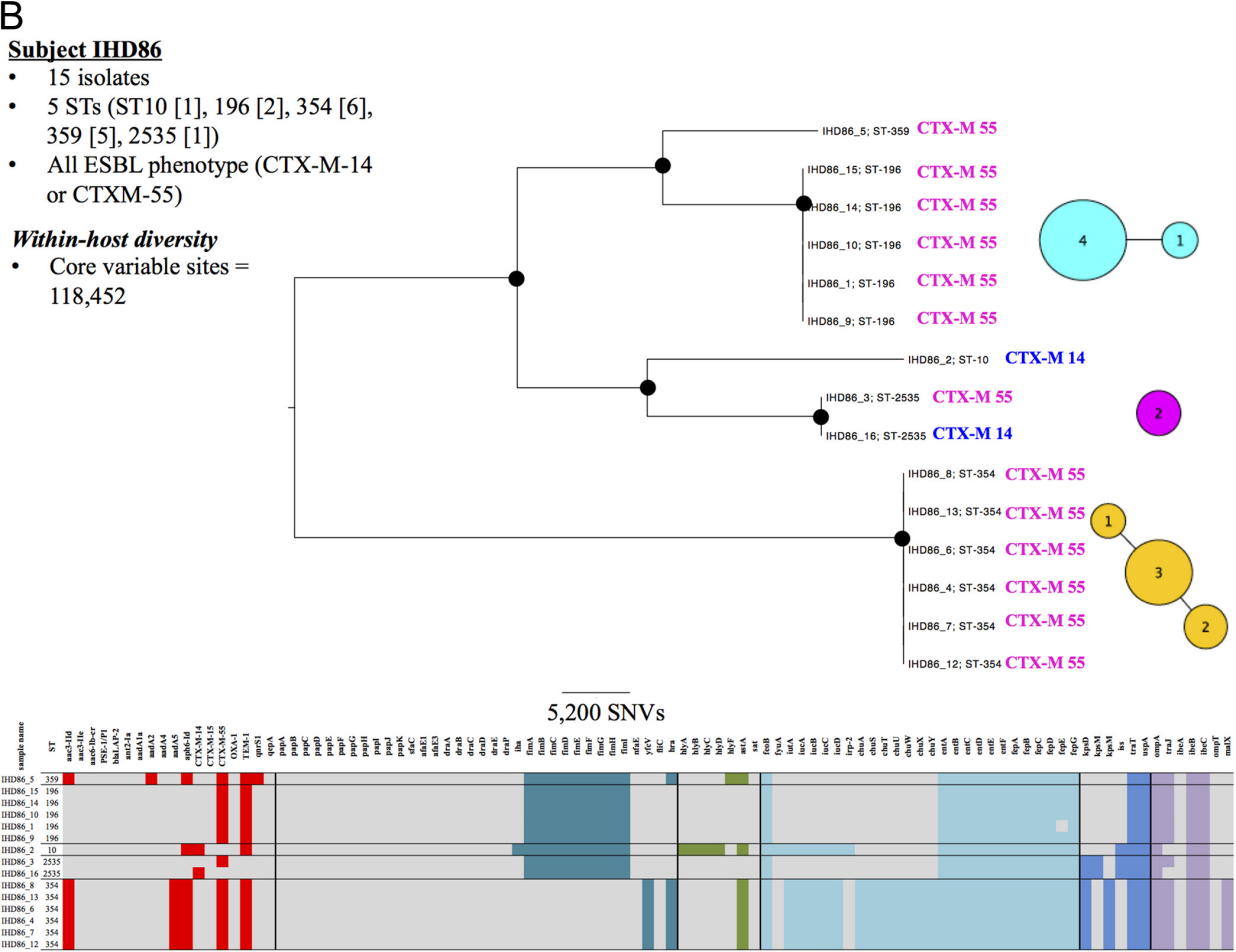

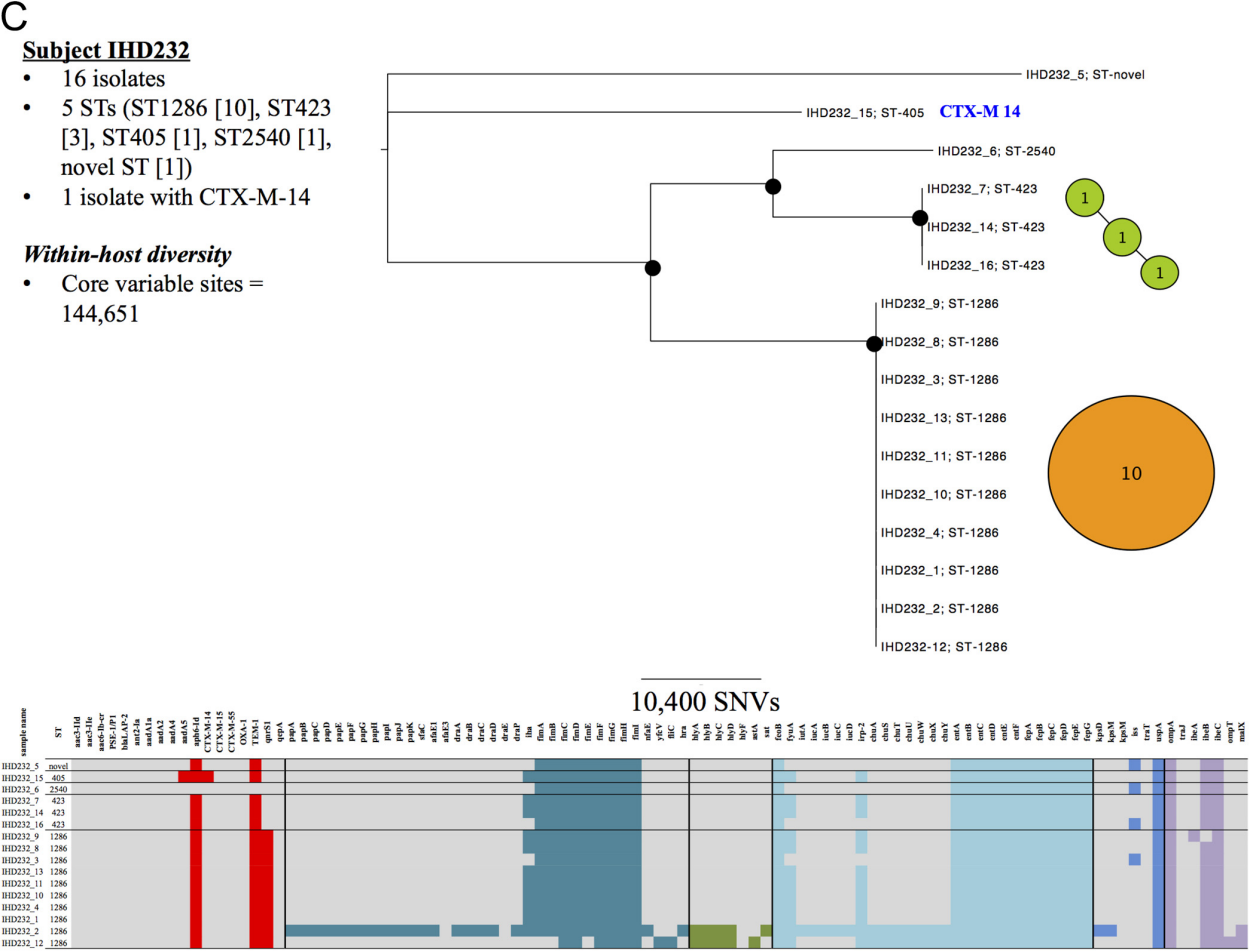

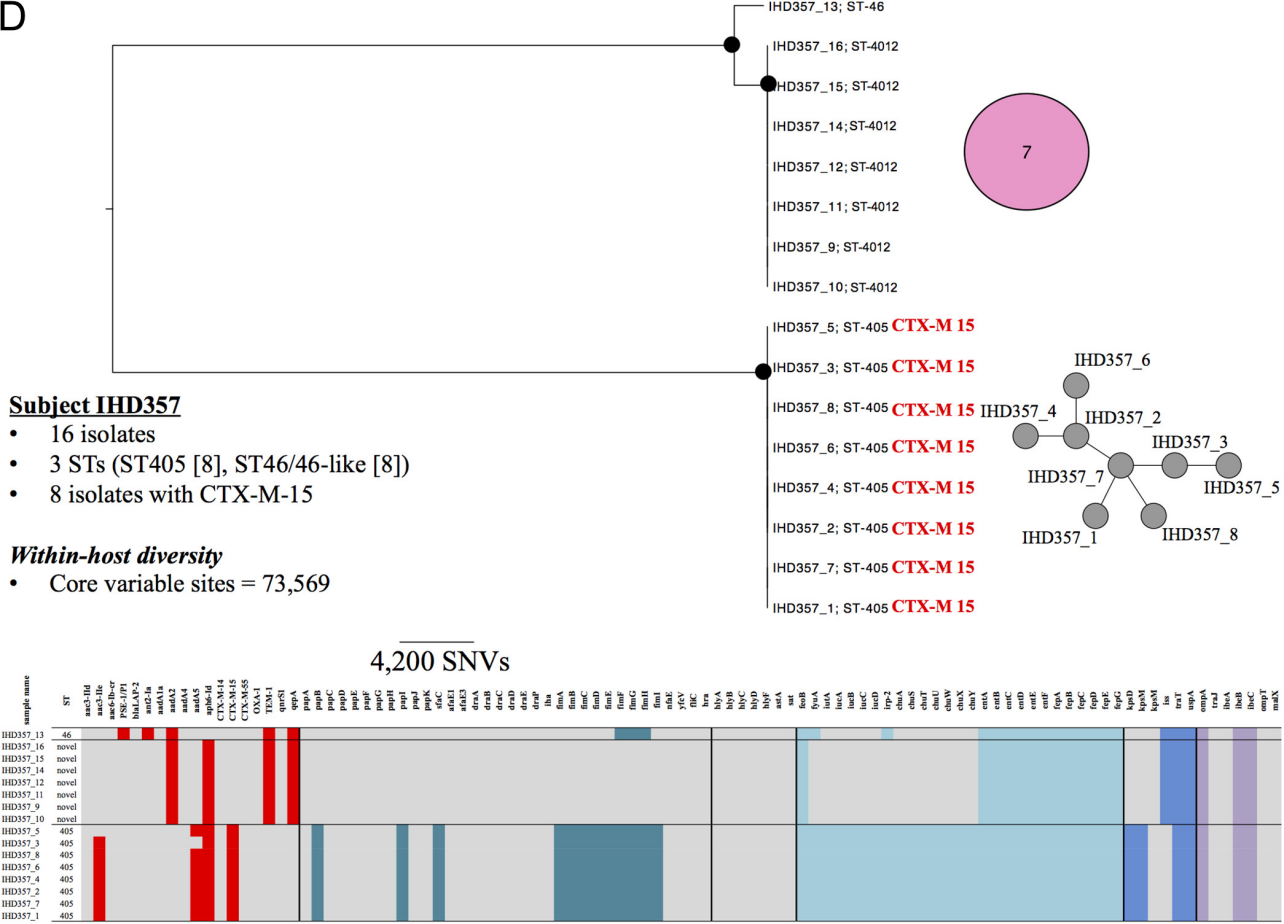

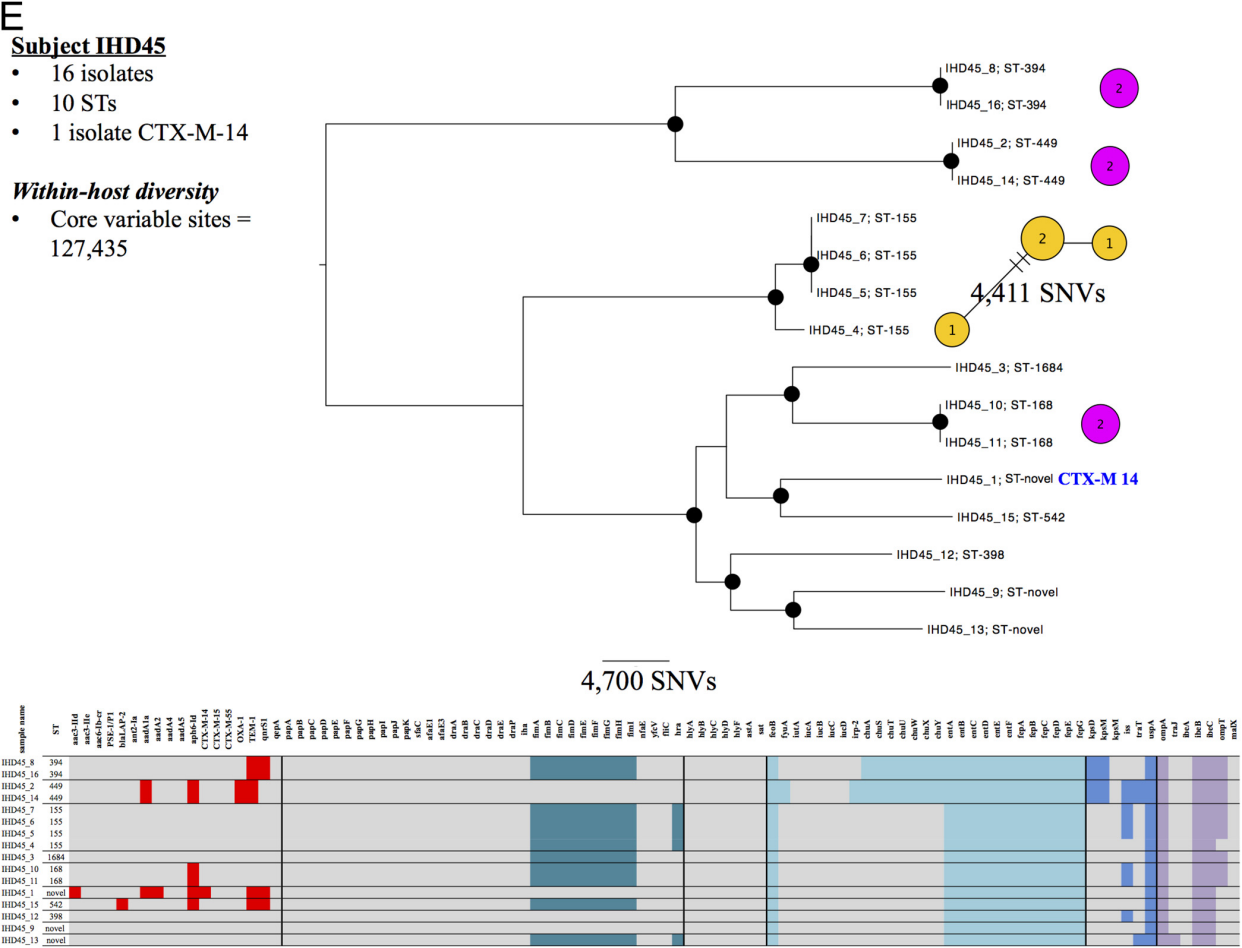
Genetic characteristics of Escherichia coli carriage isolates from subject IHD244 (A), subject IHD86 (B), subject IHD232 (C), subject IHD357 (D), and subject IHD45 (E). Black circles at tree nodes indicate bootstrap values of >95%. Colored circles represent genetic similarity of isolates within STs and are sized and labeled according to the number of isolates sharing identity. Branches between circles represent a core, single nucleotide variant. Labels adjacent to circles represent *bla*_CTX-M_ variants (colored by variant type). Within-host resistance and virulence gene profiles are represented in the heat map at the bottom of each panel, with colored blocks representing presence and gray blocks representing absence of a gene. Resistance genes are represented as red blocks; virulence factors are colored by functional category (sea green, adhesins; olive, toxins; light blue, nutritional factors; dark blue, immune evasion; lilac, miscellaneous). Isolates from within the cefpodoxime zones are labeled 1 to 8 (e.g., IHD244_1) and those from outside the zones are labeled 9 to 16 (e.g., IHD244_9), where applicable.

The number of SNVs between isolates from a single individual ranged from 1 to 144,651 (Fig. 1A to E, within-host diversity; see also Fig. S1A to C in the supplemental material). The genetic diversity observed between clones/singletons was invariably >1,000 SNVs, reflecting the extensive diversity recognized as being present in the species (29). A variety of E. coli population structures were identified in human carriers. However, only one individual carried a monoclonal population; in this instance, all 16 isolates represented a single ST (ST-10), and a single core chromosomal SNV separated one isolate from the rest. In addition, all isolates from this individual carried *bla*_CTX-M-55_ and had a homogeneous accessory component (see Fig. S1A in the supplemental material). In all other individuals, multiple STs were present, with at least one clone observed in each case.

A range of patterns was observed in the accessory genome, as represented by resistance and virulence genes. From the perspective of the clone, in total 11 clones (from seven individuals) had homogeneous accessory components, and seven clones (from five individuals) had heterogeneous accessory components. From the perspective of the individual, three individuals had clones with only homogeneous accessory components (Fig. 1A; see also Fig. S1A and B in the supplemental material), one had clones with only heterogeneous accessory components (Fig. 1C), and four had clones with both homogeneous and heterogeneous accessory components (Fig. 1B, D, and E; see also Fig. S1C).

Six of 18 clones showed observable diversity at the core genome level, in association with which the accessory genome could be either heterogeneous (Fig. 1B to E; see also Fig. S1A) or homogeneous (Fig. 1B; see also Fig. S1A). Additionally, two individuals had isolates of the same ST with substantial variation in the core genome; i.e., it exhibited within-ST SNV differences of 4,411 (Fig. 1E) and 11,315 (see Fig. S1C in the supplemental material). Notably, in the latter case, the accessory genome was invariant.

**FIG 2:**
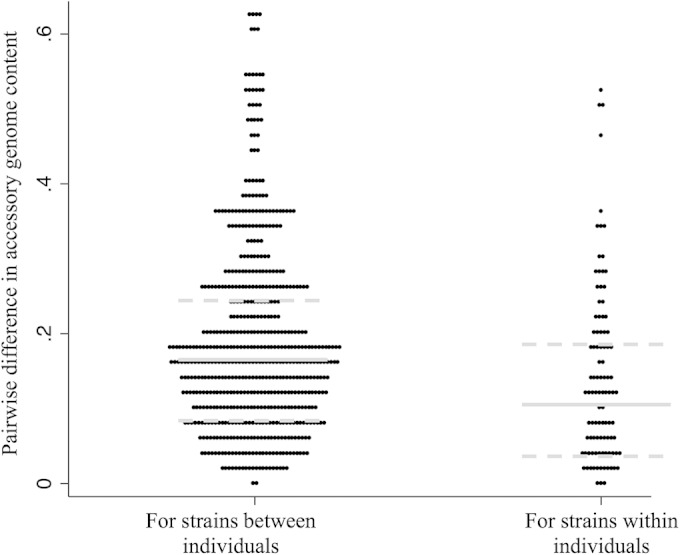
Pairwise distance comparisons of differences in the accessory gene component in clones/singletons between and within study subjects. Each dot represents a single pairwise comparison of differences in accessory gene content between each set of clones/singletons, depending on whether these strains were present in the same individual (within) or in different individuals (between). Gray lines represent the median pairwise difference for each subgroup; dotted gray lines represent the upper and lower quartiles for each subgroup.

More extensive accessory genome diversity was observed among strains recovered from different individuals than observed in strains recovered from within a single individual (Fig. 2). Based on pairwise comparisons of accessory gene content in strains, the median pairwise interstrain difference was 0.17 (IQR, 0.09 to 0.25) between individuals versus 0.11 (IQR, 0.04 to 0.19) within individuals (*P* = 0.0001).

## DISCUSSION

### Significance of the study and implications for transmission studies of intestinally carried CTX-M-positive E. coli.

This work represents, to our knowledge, the first WGS-based analysis of within-host fecal diversity of E. coli. Given the increasing importance of antimicrobial resistance in E. coli, we chose to focus on sampling individuals in a region where ESBL-producing E. coli is endemic. Although we sampled only a small group of human hosts (*n =* 8 individuals) from a single geographic location, the extent of diversity observed among ESBL-producing E. coli isolates was substantial. In addition, this diversity existed at multiple levels, as follows: (i) at the isolate level (Fig. 1E) (up to 10 STs present when 16 colonies are sampled); (ii) at the ST level, where the same ST was separated by large numbers of SNVs (Fig. 1E; see also Fig. S1C); (iii) the level of accessory gene content (here represented by resistance and virulence genes), with mosaicism of individual genes across a clone (Fig. 1C); and (iv) the level of resistance genes, such as *bla*_CTX-M_, with different variants present within a host or even an ST (Fig. 1B).

Because an individual may carry multiple antimicrobial-resistant genotypes, typing of a single resistant colony per host, by any method, is inadequate to rule out transmission. This may be less of an issue in countries and hospital settings where resistance prevalence is low and where ongoing dissemination of a single, resistant “outbreak” clone may be more likely. However, even if the diversity of ESBL-producing E. coli bacteria is currently restricted in such settings and if outbreaks are generally clonal, the situation may change as resistance prevalence continues to increase (8).

Here, we sequenced 16 individual colonies per fecal sample, which is highly resource intensive and, therefore, impracticable for routine surveillance. A previous study of Clostridium difficile demonstrated the successful use of sequencing mixtures of multiple colonies to estimate prevalence in fecal samples, to identify multiple-strain infection/colonization, and to type individual strains by *in silico* MLST (30). However, the expectation in that study, based on multiple-pick experiments similar to those conducted here, was that mixtures of no more than two subtypes were likely to be encountered. Given the extent of diversity identified in intestinal E. coli bacteria both here and in previous studies of Enterobacteriaceae (14), the methods used by Eyre et al. (30) would be inappropriate for these organisms in the vast majority of cases. More complex methods would be required to disentangle the complex population structures from short-read sequencing of mixed subcultures, if this is even possible.

One interesting question is what is the most appropriate denominator to use for calculating diversity in studies such as this. Our sampling frame should mean that, in principle, the isolates reflect the underlying frequency distribution of organisms present in each host, which is at least conditional on the sampling strategy, which here deliberately oversampled resistant organisms. Assuming each organism present has the same probability of being transmitted, by-isolate prevalence could be argued to reflect the transmission pressure most reliably. However, in all individuals at least 2 of the 16 sequenced colonies, and commonly appreciably more, were essentially identical at the core strain level; consequently, we also used a second denominator, clones plus singletons, to better reflect the diversity of discrete organisms present in the eight carriers.

### Limitations of the study.

An important limitation of the study was the inability to assess these isolates for the diversity of their mobile genetic elements, such as plasmids, in view of the difficulty in assembling these structures from short-read data. There is likely additional diversity at this level and/or shared plasmids within and among STs. Indeed, our analysis suggests that the across-clone similarity of E. coli accessory genomes is greater within than between individuals even when widely divergent isolates are present in a given host, supporting the hypothesis that horizontal gene transfer is occurring within individual hosts. This hypothesis could be further investigated by fully assembling plasmid populations using a long-read sequencing method, which would clarify the extent of plasmid transfer among genetically distinct strains within individuals.

We also made no specific comparisons with other typing methods, such as pulsed-field gel electrophoresis (PFGE) or RAPD analysis. However, PFGE has already been shown to have a lower typing resolution than WGS (32). Similarly, although the authors of a recent comparison of WGS with RAPD for the typing of fecal colonies concluded that RAPD analysis successfully identified isolates described as clonal by WGS (33), their definition of “clonal” was substantially less stringent than ours, allowing for up to 0.05% SNV differences across 1,776 single-copy core genes, conceivably representing several hundred SNVs and a significant evolutionary distance. The degree to which the additional resolution afforded by WGS substantively alters study findings and conclusions will ultimately depend on the specific question being addressed, but it enabled us to assay genetic variation at multiple, different levels using one assay.

While WGS represents a singular, unifying method by which to classify a range of genetic characteristics, the typing of multiple subcolonies using WGS is not an approach that is currently applicable to field use, and this study did not aim to identify an alternative, high-resolution, practicable method. Nevertheless, our findings have important implications for transmission studies of ESBL-producing E. coli in that if surveillance is dependent on characterizing only one or several resistant colony/colonies per sample for clonal background, resistance genes, other accessory traits, or some combination thereof, important transmission events could be missed. Our data highlight the need to characterize multiple resistant isolates per sample, by whatever typing method, to capture the diversity relevant to transmission of resistant strains.

## Supplementary Material

Supplemental material

## References

[1] Degener JE, Smit AC, Michel MF, Valkenburg HA, Muller L 1983 Faecal carriage of aerobic Gram-negative bacilli and drug resistance of Escherichia coli in different age groups in Dutch urban communities. J Med Microbiol 16:139–145. doi:10.1099/00222615-16-2-139. 6341595

[2] Cremet L, Bourigault C, Lepelletier D, Guillouzouic A, Juvin ME, Reynaud A, Corvec S, Caroff N 2012 Nosocomial outbreak of carbapenem-resistant Enterobacter cloacae highlighting the interspecies transferability of the *bla*_OXA-48_ gene in the gut flora. J Antimicrob Chemother 67:1041–1043. doi:10.1093/jac/dkr547. 22223227

[3] Forslund K, Sunagawa S, Kultima JR, Mende DR, Arumugam M, Typas A, Bork P 2013 Country-specific antibiotic use practices impact the human gut resistome. Genome Res 23:1163–1169. doi:10.1101/gr.155465.113. 23568836 PMC3698509

[4] Schjorring S, Krogfelt KA 2011 Assessment of bacterial antibiotic resistance transfer in the gut. Int J Microbiol 2011:312956. doi:10.1155/2011/312956. 21318188 PMC3034945

[5] Paterson DL, Bonomo RA 2005 Extended-spectrum beta-lactamases: a clinical update. Clin Microbiol Rev 18:657–686. doi:10.1128/CMR.18.4.657-686.2005. 16223952 PMC1265908

[6] Adler A, Gniadkowski M, Baraniak A, Izdebski R, Fiett J, Hryniewicz W, Malhotra-Kumar S, Goossens H, Lammens C, Lerman Y, Kazma M, Kotlovsky T, Carmeli Y, Mosar WP5 and WP2 Study Groups 2012 Transmission dynamics of ESBL-producing Escherichia coli clones in rehabilitation wards at a tertiary care centre. Clin Microbiol Infect 18:E497–E505. doi:10.1111/j.1469-0691.2012.03999.x. 22963432

[7] Kim J, Lee JY, Kim SI, Song W, Kim JS, Jung S, Yu JK, Park KG, Park YJ 2014 Rates of fecal transmission of extended-spectrum beta-lactamase-producing and carbapenem-resistant Enterobacteriaceae among patients in intensive care units in Korea. Ann Lab Med 34:20–25. doi:10.3343/alm.2014.34.1.20. 24422191 PMC3885768

[8] Woerther PL, Burdet C, Chachaty E, Andremont A 2013 Trends in human fecal carriage of extended-spectrum beta-lactamases in the community: toward the globalization of CTX-M. Clin Microbiol Rev 26:744–758. doi:10.1128/CMR.00023-13. 24092853 PMC3811232

[9] Snitkin ES, Zelazny AM, Thomas PJ, Stock F, NISC Comparative Sequencing Program Group, Henderson DK, Palmore TN, Segre JA 2012 Tracking a hospital outbreak of carbapenem-resistant Klebsiella pneumoniae with whole-genome sequencing. Sci Transl Med 4:148ra116. doi:10.1126/scitranslmed.3004129. PMC3521604 22914622

[10] Reddy P, Malczynski M, Obias A, Reiner S, Jin N, Huang J, Noskin GA, Zembower T 2007 Screening for extended-spectrum beta-lactamase-producing Enterobacteriaceae among high-risk patients and rates of subsequent bacteremia. Clin Infect Dis 45:846–852. doi:10.1086/521260. 17806048

[11] Grohs P, Podglajen I, Guerot E, Bellenfant F, Caumont-Prim A, Kac G, Tillecovidin B, Carbonnelle E, Chatellier G, Meyer G, Fagon JY, Gutmann L 2014 Assessment of five screening strategies for optimal detection of carriers of third-generation cephalosporin-resistant Enterobacteriaceae in intensive care units using daily sampling. Clin Microbiol Infect 20:O879–O886. doi:10.1111/1469-0691.12663. 24807791

[12] Murk JL, Heddema ER, Hess DL, Bogaards JA, Vandenbroucke-Grauls CM, Debets-Ossenkopp YJ 2009 Enrichment broth improved detection of extended-spectrum-beta-lactamase-producing bacteria in throat and rectal surveillance cultures of samples from patients in intensive care units. J Clin Microbiol 47:1885–1887. doi:10.1128/JCM.01406-08. 19386837 PMC2691084

[13] Singh K, Mangold KA, Wyant K, Schora DM, Voss B, Kaul KL, Hayden MK, Chundi V, Peterson LR 2012 Rectal screening for Klebsiella pneumoniae carbapenemases: comparison of real-time PCR and culture using two selective screening agar plates. J Clin Microbiol 50:2596–2600. doi:10.1128/JCM.00654-12. 22622443 PMC3421488

[14] Lautenbach E, Bilker WB, Tolomeo P, Maslow JN 2008 Impact of diversity of colonizing strains on strategies for sampling Escherichia coli from fecal specimens. J Clin Microbiol 46:3094–3096. doi:10.1128/JCM.00945-08. 18650357 PMC2546773

[15] Lescat M, Clermont O, Woerther PL, Glodt J, Dion S, Skurnik D, Djossou F, Dupont C, Perroz G, Picard B, Catzeflis F, Andremont A, Denamur E 2013 Commensal Escherichia coli strains in Guiana reveal a high genetic diversity with host-dependent population structure. Environ Microbiol Rep 5:49–57. doi:10.1111/j.1758-2229.2012.00374.x. 23757130

[16] Golubchik T, Batty EM, Miller RR, Farr H, Young BC, Larner-Svensson H, Fung R, Godwin H, Knox K, Votintseva A, Everitt RG, Street T, Cule M, Ip CL, Didelot X, Peto TE, Harding RM, Wilson DJ, Crook DW, Bowden R 2013 Within-host evolution of Staphylococcus aureus during asymptomatic carriage. PLoS One 8:e61319. doi:10.1371/journal.pone.0061319. 23658690 PMC3641031

[17] Young BC, Wilson DJ 2012 On the evolution of virulence during Staphylococcus aureus nasal carriage. Virulence 3:454–456. doi:10.4161/viru.21189. 23076245 PMC3485985

[18] Stoesser N, Moore CE, Golubchik T, Parry CM, Day NP, Sheppard A, Giess A, Peto TE, Walker AS, Crook DW 2014 Wide intrahost-diversity in bacterial genomes, antimicrobial resistance and virulence genes in faecally carried Escherichia coli identified using whole-genome sequencing, poster P1158 Abstr 24th Eur Soc Clin Microbiol Infect Dis, Barcelona, Spain, 10 to 13 May 2014 http://2014.eccmid.org/fileadmin/eccmid/2014/media/images/PDF_Documents/912199_ECCMID2014_LR24.pdf.

[19] Acha SJ, Kuhn I, Mbazima G, Colque-Navarro P, Mollby R 2005 Changes of viability and composition of the Escherichia coli flora in faecal samples during long time storage. J Microbiol Methods 63:229–238. doi:10.1016/j.mimet.2005.04.024. 15979748

[20] Eyre DW, Cule ML, Wilson DJ, Griffiths D, Vaughan A, O'Connor L, Ip CL, Golubchik T, Batty EM, Finney JM, Wyllie DH, Didelot X, Piazza P, Bowden R, Dingle KE, Harding RM, Crook DW, Wilcox MH, Peto TE, Walker AS 2013 Diverse sources of C. difficile infection identified on whole-genome sequencing. N Engl J Med 369:1195–1205. doi:10.1056/NEJMoa1216064. 24066741 PMC3868928

[21] Gladman S, Seemann T 2008 VelvetOptimiser, 2.1.7. Monash University, Victoria, Australia.

[22] Zerbino DR, Birney E 2008 Velvet: algorithms for de novo short read assembly using de Bruijn graphs. Genome Res 18:821–829. doi:10.1101/gr.074492.107. 18349386 PMC2336801

[23] Wirth T, Falush D, Lan R, Colles F, Mensa P, Wieler LH, Karch H, Reeves PR, Maiden MC, Ochman H, Achtman M 2006 Sex and virulence in Escherichia coli: an evolutionary perspective. Mol Microbiol 60:1136–1151. doi:10.1111/j.1365-2958.2006.05172.x. 16689791 PMC1557465

[24] Stoesser N, Batty EM, Eyre DW, Morgan M, Wyllie DH, Del Ojo Elias C, Johnson JR, Walker AS, Peto TE, Crook DW 2013 Predicting antimicrobial susceptibilities for Escherichia coli and Klebsiella pneumoniae isolates using whole genomic sequence data. J Antimicrob Chemother 68:2234–2244. doi:10.1093/jac/dkt180. 23722448 PMC3772739

[25] Chen L, Xiong Z, Sun L, Yang J, Jin Q 2012 VFDB 2012 update: toward the genetic diversity and molecular evolution of bacterial virulence factors. Nucleic Acids Res 40:D641–D645. doi:10.1093/nar/gkr989. 22067448 PMC3245122

[26] Johnson JR, Russo TA 2005 Molecular epidemiology of extraintestinal pathogenic (uropathogenic) Escherichia coli. Int J Med Microbiol 295:383–404. doi:10.1016/j.ijmm.2005.07.005. 16238015

[27] Kohler CD, Dobrindt U 2011 What defines extraintestinal pathogenic Escherichia coli? Int J Med Microbiol 301:642–647. doi:10.1016/j.ijmm.2011.09.006. 21982038

[28] Guindon S, Delsuc F, Dufayard JF, Gascuel O 2009 Estimating maximum likelihood phylogenies with PhyML. Methods Mol Biol 537:113–137. doi:10.1007/978-1-59745-251-9_6. 19378142

[29] Touchon M, Hoede C, Tenaillon O, Barbe V, Baeriswyl S, Bidet P, Bingen E, Bonacorsi S, Bouchier C, Bouvet O, Calteau A, Chiapello H, Clermont O, Cruveiller S, Danchin A, Diard M, Dossat C, Karoui ME, Frapy E, Garry L, Ghigo JM, Gilles AM, Johnson J, Le Bouguenec C, Lescat M, Mangenot S, Martinez-Jehanne V, Matic I, Nassif X, Oztas S, Petit MA, Pichon C, Rouy Z, Ruf CS, Schneider D, Tourret J, Vacherie B, Vallenet D, Medigue C, Rocha EP, Denamur E 2009 Organised genome dynamics in the Escherichia coli species results in highly diverse adaptive paths. PLoS Genet 5:e1000344. doi:10.1371/journal.pgen.1000344. 19165319 PMC2617782

[30] Eyre DW, Cule ML, Griffiths D, Crook DW, Peto TE, Walker AS, Wilson DJ 2013 Detection of mixed infection from bacterial whole genome sequence data allows assessment of its role in Clostridium difficile transmission. PLoS Comput Biol 9:e1003059. doi:10.1371/journal.pcbi.1003059. 23658511 PMC3642043

[31] Reeves PR, Liu B, Zhou Z, Li D, Guo D, Ren Y, Clabots C, Lan R, Johnson JR, Wang L 2011 Rates of mutation and host transmission for an Escherichia coli clone over 3 years. PLoS One 6:e26907. doi:10.1371/journal.pone.0026907. 22046404 PMC3203180

[32] Price LB, Johnson JR, Aziz M, Clabots C, Johnston B, Tchesnokova V, Nordstrom L, Billig M, Chattopadhyay S, Stegger M, Andersen PS, Pearson T, Riddell K, Rogers P, Scholes D, Kahl B, Keim P, Sokurenko EV 2013 The epidemic of extended-spectrum-beta-lactamase-producing Escherichia coli ST131 is driven by a single highly pathogenic subclone, H30-Rx. mBio 4(6):e00377-13. doi:10.1128/mBio.00377-13. PMC3870262 24345742

[33] Nielsen KL, Godfrey PA, Stegger M, Andersen PS, Feldgarden M, Frimodt-Moller N 2014 Selection of unique Escherichia coli clones by random amplified polymorphic DNA (RAPD): Evaluation by whole genome sequencing. J Microbiol Methods 103:101–103. doi:10.1016/j.mimet.2014.05.018. 24912108 PMC4166437

