# Supplementary material for: Extensive Within-Host Diversity in Fecally Carried Extended-Spectrum-Beta-Lactamase-Producing Escherichia coli Isolates: Implications for Transmission Analyses
Source: J Clin Microbiol. 2015 Jun 18;53(7):2122–31. doi: 10.1128/JCM.00378-15 (PMC4473215; doi:10.1128/JCM.00378-15)
Supplement: Supplemental material [file supp_53_7_2122__index.html]

Supplemental material 

# Extensive within-host diversity in fecally carried extended-spectrum beta-lactamase-producing *Escherichia coli*: implications for transmission analyses

## Supplemental material

- Supplemental file 1 -

  Fig. S1 (Genetic characteristics of *Escherichia coli* carriage isolates from subjects IHD813, IHD1178, and IHD717)

  PDF, 445K
- Supplemental file 2 -

  Table S1 (Summary of sequenced strains)

  XLS, 85K
