# Supplementary material for: Extensive Within-Host Diversity in Fecally Carried Extended-Spectrum-Beta-Lactamase-Producing Escherichia coli Isolates: Implications for Transmission Analyses
Source: J Clin Microbiol. 2015 Jun 18;53(7):2122–31. doi: 10.1128/JCM.00378-15 (PMC4473215; doi:10.1128/JCM.00378-15)

Supplementary Figure 1. Genetic characteristics of *Escherichia coli* carriage isolates from A. subject IHD813; B. subject IHD1178; C. subject IHD717. Black circles at tree nodes indicate bootstrap values of > 95%. Colored circles represent genetic similarity of isolates within STs, and are sized and labeled according to the number of isolates sharing identity. Branches between circles represent a core, single nucleotide variant. Labels adjacent to circles represent *bla*$_{CTX-M}$ variants (colored by variant type). Within-host resistance and virulence gene profiles for all isolates in this individual are represented in the heatmap at the bottom of each panel, with colored blocks representing presence, and gray blocks absence of a gene. Resistance genes are represented as red blocks; virulence factors are colored by functional category (sea-green = adhesins; olive = toxins; light blue = nutritional factors; dark blue = immune evasion; lilac = miscellaneous). Isolates from within the cefpodoxime zones are labelled _1-8; from outside the zones _9-16, where applicable.

Subject IHD813

- 16 isolates
- Same ST (ST10)
- All ESBL phenotype (all CTX-M-55)

Within-host diversity

- Single core SNV difference between isolates

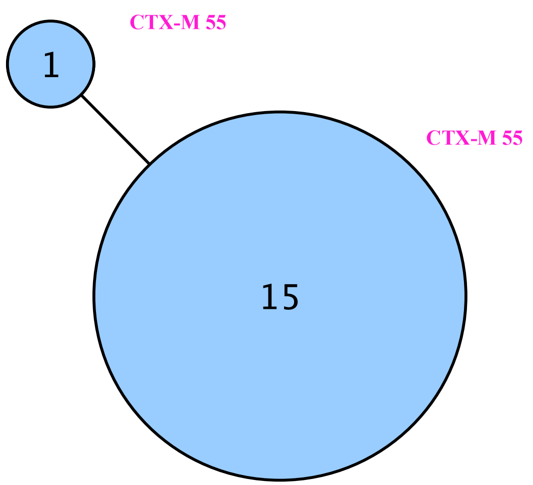

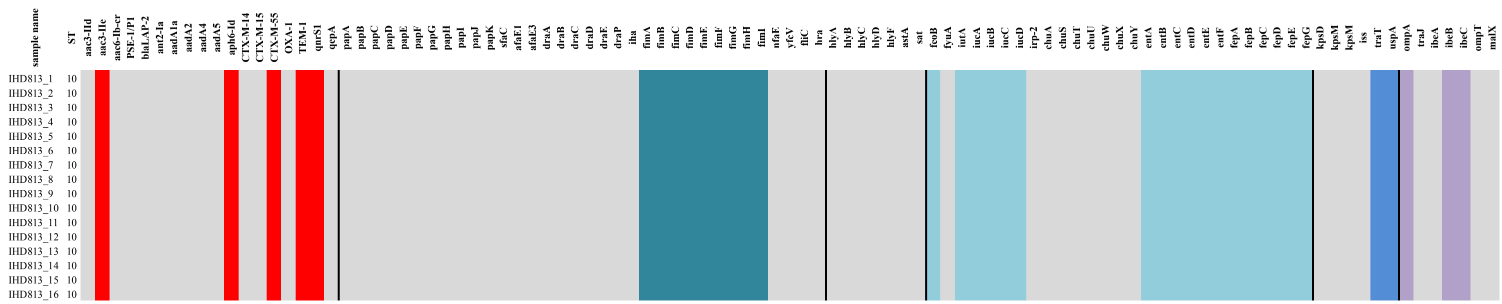

Subject IHD1178

- 16 isolates
- 3 STs (ST648 [13], ST405 [2], novel ST [1])
- 16 isolates with CTX-M-15

Within-host diversity

- Core variable sites = 110,865

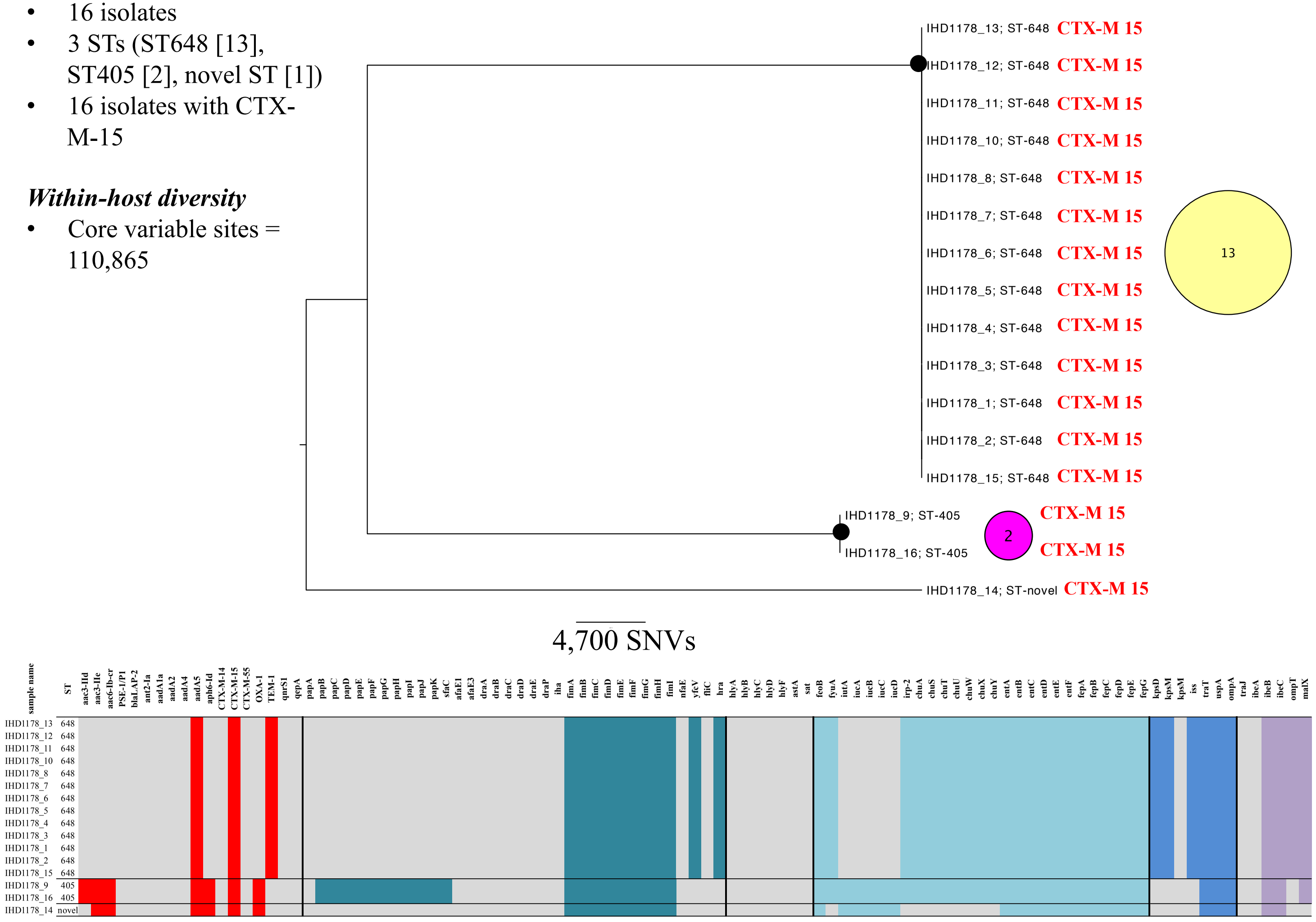

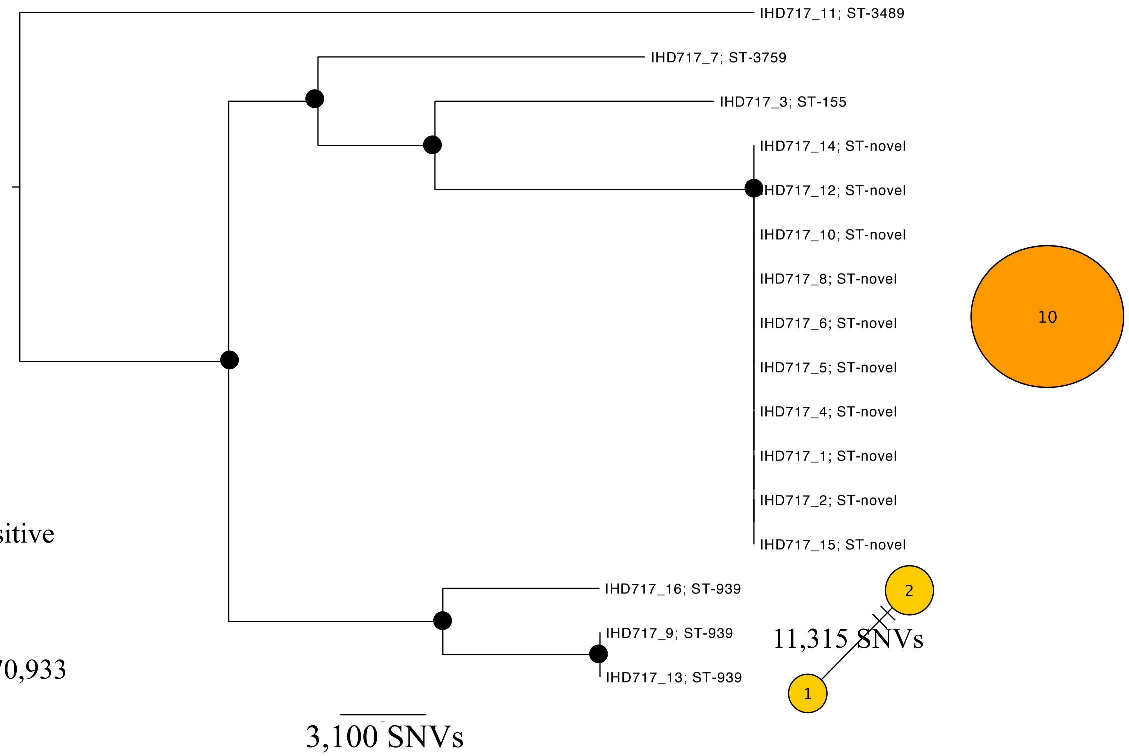

Subject IHD717

- 16 isolates
- 5 STs
- No CTX-M/ESBL positive isolates

Within-host diversity

- Core variable sites = 70,933

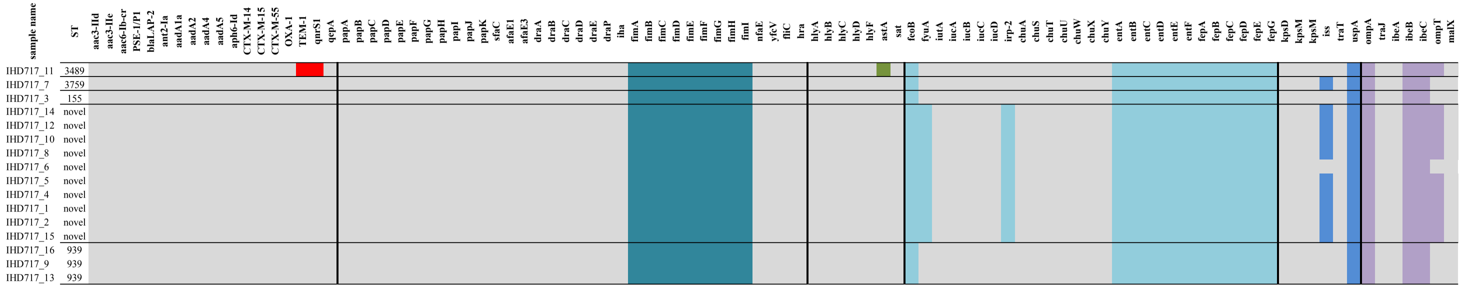

Supplement: Supplemental material [file JCM.00378-15_zjm999094325so1.pdf]
